# Epithelial cell adhesion molecule (EpCAM) is involved in prostate cancer chemotherapy/radiotherapy response in vivo

**DOI:** 10.1186/s12885-018-5010-5

**Published:** 2018-11-12

**Authors:** Jie Ni, Paul Cozzi, Julia Beretov, Wei Duan, Joseph Bucci, Peter Graham, Yong Li

**Affiliations:** 1 Cancer Care Centre, St George Hospital, Level 2, 4-10 South St, Kogarah, NSW 2217 Australia; 2 St George and Sutherland Clinical School, Faculty of Medicine, UNSW Sydney, Kensington, NSW 2052 Australia; 3 Department of Surgery, St George Hospital, Kogarah, NSW 2217 Australia; 4 Anatomical Pathology, NSW Health Pathology, St George Hospital, Gray St, Kogarah, NSW 2217 Australia; 5 School of Medicine and Centre for Molecular and Medical Research, Deakin University, Waurn Ponds, VIC 3216 Australia; 6 School of Basic Medical Sciences, Zhengzhou University, Henan, 450001 China

**Keywords:** EpCAM, Prostate cancer, Animal model, PI3K/Akt/mTOR signaling pathway, Chemoresistance, Radioresistance

## Abstract

**Background:**

Development of chemo−/radioresistance is a major challenge for the current prostate cancer (CaP) therapy. We have previously demonstrated that epithelial cell adhesion molecule (EpCAM) is associated with CaP growth and therapeutic resistance in vitro, however, the role of EpCAM in CaP in vivo is not fully elucidated. Here, we aimed to investigate how expression of EpCAM is involved in CaP growth and chemo−/radiotherapy response in NOD/SCID mouse models in vivo and to validate its role as a therapeutic target for CaP therapy.

**Methods:**

EpCAM was knocked down in PC-3 CaP cell line using short hairpin RNA (shRNA). The effect of EpCAM-knockdown (KD) on tumour growth, chemo−/radiotherapy response and animal survival was evaluated on subcutaneous (s.c) and orthotopic mouse models.

**Results:**

We found that KD of EpCAM significantly inhibited tumour growth, increased xenograft sensitivity to chemotherapy/radiotherapy, and prolonged the survival of tumour-bearing mice. In addition, we demonstrated that KD of EpCAM is associated with downregulation of the PI3K/Akt/mTOR pathway.

**Conclusions:**

In conclusion, our data confirms that CaP growth and chemo−/radioresistance in vivo is associated with over-expression of EpCAM, which serves both a functional biomarker and promising therapeutic target.

**Electronic supplementary material:**

The online version of this article (10.1186/s12885-018-5010-5) contains supplementary material, which is available to authorized users.

## Background

Prostate cancer (CaP) is the commonly diagnosed and the second leading cause of cancer-related death in males in the USA in 2017 [1]. Thanks to the radical prostatectomy and radiotherapy (RT) patients with localized CaP can achieve excellent long-term survival. However, for recurrent and metastatic diseases, while initially responsive to androgen deprivation therapy, recurrent castration-resistant prostate cancer (CRPC) will inevitably occur, and is often associated with a poor prognosis.

RT continues to be one of standard treatment options for the different stages of CaP. Despite recent advances in the approaches of delivering radiation to the prostate, around 30–50% of patients who undergo RT will experience biochemical recurrence within 10 years following treatment [2], which highlights the importance of understanding the biological mechanisms underlying radioresistance in attempts to predict patient response to RT prior to treatment and to identify novel therapeutic targets to overcome CaP radioresistance.

Despite the revolutionary targeted therapies and immunotherapy in CaP management, chemotherapy retains a key role in the treatment of advanced CaP disease. Several recent clinical trials showed profound benefits with docetaxel (DTX) by prolonging the survival, at the cost of significant toxicity [3, 4]. DTX plus prednisone regimens has been the first-line chemotherapy since 2014, however, not much progress has been made in an effort to further improve the outcome for CRPC patients [5]. Hence, it is important to uncover the key mechanisms of DTX resistance of CaP and identify effective therapeutic targets to improve current chemotherapeutic modalities.

Epithelial cell adhesion molecule (EpCAM), also known as CD326, is a transmembrane glycoprotein that is highly expressed in rapidly proliferating carcinomas [6]. Recent data suggest that EpCAM plays an important role not only in cell-cell adhesion, but also in cell signalling, migration, proliferation and differentiation [7]. Our previous study also demonstrated that over-expression of EpCAM was found in primary CaP tissues and lymph node metastases and is positively associated with CaP metastasis and therapeutic resistance in vitro [8]. However, little work has been done to elucidate the roles of EpCAM in CaP chemo−/radioresistance in vivo. To address this unmet need, in the current study, the effect of EpCAM on CaP growth and chemo−/radiotherapy response was assessed by testing the tumor growth rate of CaP subcutaneous (s.c.) and orthotopic xenografts on NOD/SCID mice. We demonstrate for the first time that EpCAM is involved in tumour growth, chemo−/radiotherapy response and associated with activation of the PI3K/Akt/mTOR signalling pathway in CaP animal models in vivo*.* Our results suggest that EpCAM is not only an important functional biomaker in CaP development and therapeutic sensitivity, but also a promising therapeutic target to repress CaP growth and overcome the resistance to chemo−/radiotherapy.

## Materials

### Antibodies and reagents

Detailed information and conditions for all antibodies are listed in Additional file 1: Table S1. DTX was purchased from Hospira Australia Pty Ltd. (Melbourne, VIC, Australia). For in vitro study, DTX was first diluted in 100% ethanol and then added to the growth medium. For in vivo study, DTX was diluted in 0.9% saline.

### Cell line and cell culture

PC-3 CaP cell line was purchased from American Type Culture Collection (Rockville, MD, USA) and cultured in RPMI-1640 supplemented with 10% (vol/vol) fetal bovine serum (FBS), 50 U/mL of penicillin, and 50 μg/mL of streptomycin. All cell culture reagents were supplied by Life Technologies Australia Pty Ltd. (Mulgrave, VIC, Australia) unless otherwise stated. The cell line was maintained in a humidified incubator at 37 °C and 5% CO_2_.

The identity of cell lines was confirmed by short tandem repeat profiling and experiments were carried out within a limited number of passages of initial authentication.

### shRNA transfection for EpCAM

Knockdown (KD) of EpCAM were achieved using a previously published method with modification [9]. Three MISSION® lentiviral transduction particles encoding for shRNA against EpCAM and MISSION® non-target shRNA control transduction particles were used (Sigma-Aldrich, Pty Ltd., Castle Hills, NSW, Australia). After transfection, the stable clones were selected in cell culture medium containing 0.5 mg/mL puromycin (Invitrogen Australia Pty Ltd., Melbourne, VIC, Australia), and propagated for the following experiments.

### Immunofluorescence (IF) staining

Cells grown on Millicell® EZ slides (Merck Millipore, VIC, Australia) were fixed by cold methanol, rinsed by TBS (pH 7.5), blocked in 10% (vol/vol) goat serum, and incubated with rabbit anti-human EpCAM antibody at 4 °C overnight (o/n), and then incubated with Alexa Fluro® 488 goat anti-rabbit IgG (H + L) secondary antibody for 1 h at room temperature (r/t). Negative controls were treated identically, but using non-specific rabbit IgG. Propidium iodide (PI) was used for nuclei staining.

Immunofluorescence staining was then visualised using a ZEISS Axio Vert.A1 microscope (Carl Zeiss Microscopy, Germany).

### Western blot

Western blot analysis was performed as previously described [9]. Briefly, 20 μg whole cell lysates were separated on a Bis-Tris gel and then transferred to a polyvinylidene difluoride (PVDF) membrane. The membrane was blocked with 5% bovine serum albumin (BSA) in Tris-buffered saline with 0.1% Tween20 (TBS-T), and then incubated with primary antibodies (Additional file 1: Table S1) at 4 °C o/n. Following incubation with HRP-conjugated secondary antibodies, immunoblot was analysed using SuperSignal West Pico enhanced chemiluminescence (ECL) substrate (Pierce Chemical Co, Rockford, USA) [9]. Mouse anti-human β-tubulin monoclonal antibody (MAb) was used as a loading control.

### Quantitative real-time PCR (qRT-PCR)

Total RNA was isolated with High Pure RNA isolation kit (Roche Life Science, NSW, Australia) and 2 μg of total RNA was used to synthesise cDNA using the SuperScript III First-strand Synthesis System Kit (Invitrogen Pty Ltd., VIC, Australia), according to the manufacturers’ protocol. qRT-PCR was carried out on a Rotor-Gene instrument (Corbett Life Science, NSW, Australia) in a solution containing SYBR® Select Master Mix (Life Technologies Pty Ltd., VIC, Australia), primer and cDNA in a final volume of 20 μL. GAPDH was used as a reference control.

### Experimental animals

All animal care and experimentation procedures followed the guideline according to protocols approved by the Animal Care and Ethics Committee (ACEC 14/46A) of UNSW Sydney and conformed to the Animal Research Act 1985 and Australian code for the care and use of animals for scientific purposes 8th edition (2013).

Male, 6–8 weeks old NOD/SCID mice of a weight of 15-20 g (Animal Resources Centre, WA, Australia) were housed in a group of 3 or 4, in specific pathogen-free facilities and all experiments were performed in a laminar flow cabinet in the morning of the day unless stated otherwise. House and husbandry conditions were a 12:12 light:dark cycle, mouse maintenance food and water. Environmental enrichment included bedding, one red tinted guinea pig hut and one handful of paper wool.

Mice were kept at least 1 week before experimental manipulation. All mice were monitored daily for health status and weight were recorded at least twice a week. All mice remained healthy and active during the experiments.

### Establishment of s.c. and orthotopic xenograft animal models

To establish s.c. xenograft model, as described previously [9, 10], mice were randomised into two groups, and 2 × 10^6^ PC-3-EpCAM-KD or PC-3-EpCAM-scr CaP cells in 100 μL Dulbecco’s phosphate-buffered saline (DPBS) were injected subcutaneously in the right rear flank region of mice, respectively. Based on our previous study and a Power calculation, tumour progression of 10 mice in each cohort (*n* = 10) was measured weekly using callipers, and tumour volumes were calculated as 0.5 × (length × width^2^) (in millimetres) for up to 8 weeks. Upon sacrifice, tumour xenografts were harvested for histological examination and immunohistochemistry (IHC).

To establish orthotopic xenograft model, mice were randomised into two groups, and 1 × 10^6^ PC-3-EpCAM-KD or PC-3-EpCAM-scr CaP cells suspended in 50 μL DPBS were injected into the prostatic lobe after exposure through a lower midline laparotomy incision. Starting from the second week after cell inoculation, based on our previous study and a Power calculation, tumour progression of mice in each cohort (*n* = 10) was monitored weekly by 3D ultrasound as described in the previous publication [11]. Upon sacrifice, primary tumours were harvested for histological examination and IHC.

### DTX toxicity study in NOD/SCID mice without tumours

The dose-tolerance relationship was examined in 6 NOD/SCID mice/per group without tumours following a single i.p. administration of DTX, or saline as VC treatment respectively to determine the maximum tolerance dose (MTD). The candidate doses of DTX were 10, 20, 30, 40, 50 mg/kg, based on our previous publication [9]. The MTD was the dose at one level below when 1/3 of the mice reach the endpoints that were defined in the monitoring criteria.

### DTX response in two CaP animal models

When average tumour size reached 70 ± 10 mm^3^ in s.c. model in each subgroup, 4 mice/per group (*n* = 4) including PC-3-EpCAM-KD and PC-3-EpCAM-scr cell lines were treated with 50 mg/kg DTX (MTD) once by i.p. injection or with VC (saline), respectively. Drugs and vehicle administration was randomised. Tumour growth was plotted by measurements using callipers as previously published [9].

When average tumour size reached 50 ± 10 mm^3^ in orthotopic model in each subgroup, 4 mice/per group (*n* = 4) including PC-3-EpCAM-KD and PC-3-EpCAM-scr cell lines were treated with 50 mg/kg DTX once by i.p. injection or with VC (saline), respectively, based on the pilot study. Drugs and vehicle administration was randomised. Tumour growth was monitored by ultrasonography and calculated using Vevo® Lab 1.7.0 software (VisualSonics Inc., Ontario, Canada) according to our previously published method [11].

### Radiation tolerance study in NOD/SCID mice without tumours

The tolerance study was also performed to determine the MTD in mice for RT. The dose-tolerance relationship was examined in NOD/SCID mice without tumours for local radiation treatment. Mice were anaesthetised with 50–80 mg/kg ketamine and 3–5 mg/kg xylazine (i.p.) and immobilised in a custom-designed chamber with a lead shield covering the upper half of their bodies. Mice were irradiated in the X-RAD 320 Biological Irradiator (Precision X-Ray, North Branford, CT, USA) with only lower abdomen areas being exposed. Two mice were used in each group. Mice in each group received a dose of 2Gy/day for 2, 3, 4 and 5 consecutive days (accumulatively 4, 6, 8, 10 Gy), respectively. A sham control (SC) radiation group was also included. The MTD for RT chosen for subsequent studies was the highest dose without the exhibition of endpoints that were defined in the animal monitoring criteria.

### Radiation response in two CaP animal models

When average tumour size reached 70 ± 10 mm^3^ in s.c. model in each subgroup, 4 mice/per group (*n* = 4) including PC-3-EpCAM-KD and PC-3-EpCAM-scr cell lines were irradiated at a dose of 2 Gy for 4 consecutive days (a total of 8Gy) or while 4 control mice/per group (*n* = 4) went through the same procedure with the SC irradiation. Irradiation and sham irradiation exposure was randomised.

When average tumour size reached 50 ± 10 mm^3^ in orthotopic model in each subgroup, 4 mice/per group (*n* = 4) including PC-3-EpCAM-KD and PC-3-EpCAM-scr cell lines were irradiated at a dose of 2 Gy for 4 consecutive days (a total of 8 Gy) or with SC (no irradiation). Irradiation and sham irradiation exposure was randomised.

### Mouse survival study in s.c model

NOD/SCID mouse s.c. xenograft model was established using PC-3-EpCAM-KD or PC-3-EpCAM-scr CaP cells as described above. When tumour size reached 70 ± 10 mm^3^, 6 mice/per group (*n* = 6) received no treatment (used as control), DTX treatment (50 mg/kg, i.p.), DTX-VC treatment (saline, i.p.) radiation (2 Gy per day for 4 consecutive days, a total dose of 8 Gy) or RT-SC treatment (no radiation), respectively. Allocation of mice into each cohort was randomised.

Tumour size was documented weekly using a calliper for up to 13 weeks. Endpoints were defined in the animal monitoring criteria. Comparison of the survival of mice was made using a Kaplan-Meier curve.

### IHC

Standard immunohistochemistry procedures were used to visualise biomarkers of interest as previously published [12]. Briefly, paraffin sections were de-parrafinised and rehydrated, then incubated with primary antibodies o/n at 4 °C. Slides were then incubated with HRP-conjugated secondary antibody (1:150 dilution) for 45 min at r/t, followed by 3,3′ diaminobenzidine (DAB) substrate solution (Sigma-Aldrich, Pty Ltd., Castle Hill, NSW, Australia) containing 0.03% hydrogen peroxide (VWR International, QLD, Australia), Harris Hematoxylin (Thermo Fisher Pty Ltd., VIC, Australia) for counter-staining and Scott’s bluing solution (Sigma-Aldrich Pty Ltd., NSW, Australia) for bluing. Tissue sections were washed in water, dehydrated, cleared and mounted.

### Assessment of immunostaining

Staining intensity (0–3) was assessed using a light microscopy (Leica, Germany). The assessment criteria were as previously reported [13], where: 0 (negative, 0–10%); 1+ (weak, 10–45%); 2+ (moderate, 45–70%); 3+ (strong, 70%) of the tumour cells stained. Blind scoring was performed independently by three experienced observers (JN, JB and YL), and the average of grades was recorded.

### Statistical analysis

All numerical data were expressed as mean ± standard deviation (SD). Data from two different groups were analysed using two-tail Student’s t-test. All *P* values were 2-sided. *P* < 0.05 was considered significant. The Kaplan-Meier method and logrank test were used for endpoint survival analysis. All statistical analyses were performed using the GraphPad Prism 7.02 software (GraphPad, San Diego CA).

## Results

### KD of EpCAM in PC-3 cell line

**Fig. 1:**
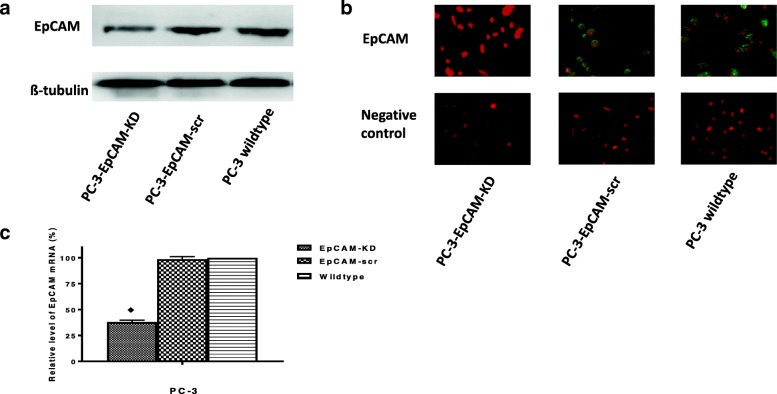
Reduced expression of EpCAM in PC-3-EpCAM-KD cell line. The knock-down effect of EpCAM was confirmed by Western blotting, immunofluorescence staining and qRT-PCR in PC-3 CaP cell line. **a** Western blotting is shown the reduced expression of EpCAM in PC-3-EpCAM-KD cell line compared with PC-3-EpCAM-scr and PC-3 wildtype cell lines. ß-tubulin was chosen as a loading control. **b** Representative immunofluorescence images of PC-3-EpCAM-KD, PC-3-EpCAM-scr and PC-3 wildtype cell lines. Green indicates positive-EpCAM staining and nuclei are stained with PI (red). Magnification: all images × 400. **c** Knock-down effects from Western blotting and immunofluorescence are further confirmed by qRT-PCR (♦ indicates *P* < 0.05). All results were from three independent experiments (mean ± SD, *n* = 3). knock-down; scr, scrambled shRNA control

As shown in Fig. 1, PC-3 CaP cells were transfected with EpCAM-shRNA or control shRNA. KD of EpCAM was confirmed at both protein and mRNA levels. Western blot (Fig. 1a) and IF staining (Fig. 1b) and qRT-PCR (Fig. 1c) showed that EpCAM expression level was markedly reduced in PC-3-EpCAM-KD cells compared with PC-3-EpCAM-scr and PC-3 cell lines. No significant difference was noted between PC3-EpCAM-scr cells and PC-3 wild-type control cells in each group (Fig. 1).

### KD of EpCAM affects tumourigenicity in PC-3 s.c. and orthotopic xenograft mouse models

**Fig. 2:**
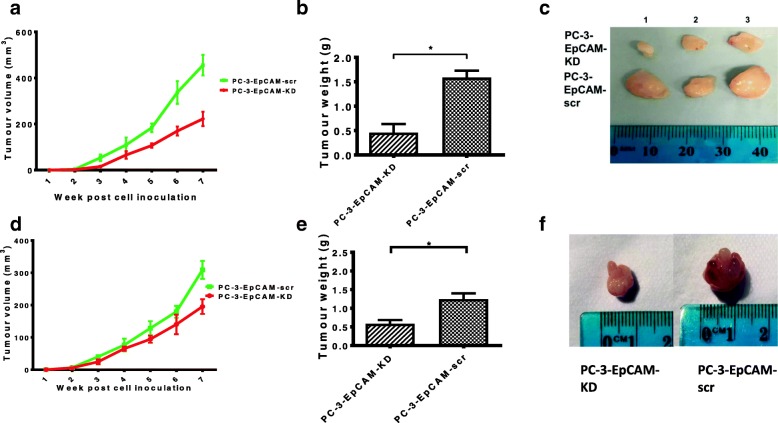
Tumour growth of PC-3-EpCAM-KD and PC-3-EpCAM-scr cells in CaP s.c. and orthotopic animal models. **a** The graph is shown for tumour growth curves of PC-3-EpCAM-KD and PC-3-EpCAM-scr in s.c. xenograft model. The weekly measurements of PC-3-EpCAM-KD xenografts show a significant reduced tumour growth rate compared to the PC-3-EpCAM-scr group (*n* = 10, mean ± SD, *P* < 0.05). **b** At the end of the experiment, tumour weight from PC-3-EpCAM-KD group mice was significantly decreased compared to that from PC-3-EpCAM-scr group mice in s.c. model (*n* = 10, mean ± SD, * indicates *P* < 0.05). **c** At the end of the experiment, representative images of tumours from s.c. xenografts are shown. **d** The graph represents for PC-3-EpCAM-KD and PC-3-EpCAM-scr tumour growth curves in the orthotopic xenograft model (*n* = 10, mean ± SD, *P* < 0.05). **e** Tumour weight from PC-3-EpCAM-KD group mice was shown as significantly decreased compared to that from PC-3-EpCAM-scr group mice in orthotopic model (n = 10, mean ± SD, * indicates *P* < 0.05). **f** Representative images of tumours from orthotopic xenografts are shown for PC-3-EpCAM-KD and PC-3-EpCAM-scr tumour xenografts. KD, knock-down; scr, scrambled shRNA control

As shown in the tumour growth graphs in Fig. 2, the weekly measurements of s.c. and orthotopic xenografts were plotted. The tumour growth curve showed a significantly reduced tumour growth rate of PC-3-EpCAM-KD xenografts compared to the PC-3-EpCAM-scr group (*n* = 10; *P* < 0.05), in both s.c. and orthotopic models, respectively.

In s.c. model, all xenograft tumours (*n* = 10) became palpable from the third week after cell inoculation in PC-3-EpCAM-KD group while all xenograft tumours (*n* = 10) became palpable from the second week after cell inoculation in PC-3-EpCAM-scr group. Significant tumour growth difference was found 3 weeks post cell inoculation (Mean ± SD: EpCAM-KD, 15.2 ± 2.2 mm^3^; EpCAM-scr, 53.2 ± 14.1 mm^3^; Fig. 2a, *P* < 0.001). After five weeks post cell inoculation, in PC-3-EpCAM-KD group, tumour volume was 106.4 ± 9.5 mm^3^, while in PC-3-EpCAM-scr group, tumour volume was 183.6 ± 18.4 mm^3^ (Mean ± SD, Fig. 2a, *P* < 0.001). At the end of experiment (7 weeks post cell inoculation), mouse tumours were harvested, measured and weighed. In PC-3-EpCAM-KD group, tumour volume was 222.3 ± 31.1 mm^3^, tumour weight was 0.5 ± 0.2 g; whereas in PC-3-EpCAM-scr group, tumour volume was 455.8 ± 44.5 mm^3^, tumour weight was 1.6 ± 0.1 g (Mean ± SD, Fig. 6b, *P* < 0.001 in both measurement), suggesting that a significant difference exists in tumour volume and weight between the two sublines. Typical images for tumour sizes of different groups of mice in s.c model at the end of experiment are shown in Fig. 2c.

In orthotopic model, 3D ultrasound imaging was used to visualise and monitor tumour growth. The prostatic tumour became visible from the second week post cell inoculation in both PC-3-EpCAM-KD and PC-3-EpCAM-scr group. Significant tumour growth difference was found 3 weeks post cell inoculation (Mean ± SD: EpCAM-KD, 24.7 ± 6.0 mm^3^; EpCAM-scr, 41.8 ± 4.3 mm^3^; Fig. 2d, *P* < 0.01). After five weeks post cell inoculation, in PC-3-EpCAM-KD group, tumour volume was 90.8 ± 11.3 mm^3^, while in PC-3-EpCAM-scr group, tumour volume was 129.7 ± 17.6 mm^3^ (Mean ± SD, Fig. 2d, *P* < 0.01). At the end of experiment (7 weeks post cell inoculation), tumours were harvested, measured and weighed. In PC-3-EpCAM-KD group, tumour volume was 187.2 ± 24.9 mm^3^, tumour weight was 0.6 ± 0.1 g; whereas in PC-3-EpCAM-scr group, tumour volume was 304.1 ± 24.2 mm^3^, tumour weight was 1.2 ± 0.2 g (Mean ± SD, Fig. 2e, *P* < 0.001 in both measurements), indicating a significant difference in tumour volume and weight in orthotopic model from the two sublines. Typical images for tumour sizes of different groups of mice in orthotopic model at the end of experiment are shown in Fig. 2f.

### KD of EpCAM increased DTX sensitivity in PC-3 s.c. and orthotopic xenograft mouse models

**Fig. 3:**
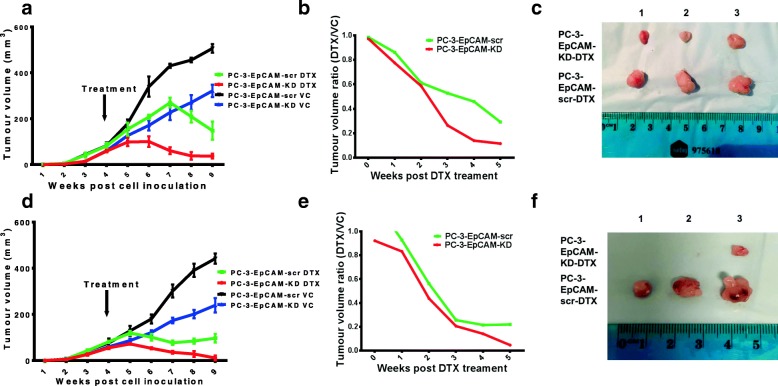
In vivo effects of EpCAM-KD on chemosensitivity in s.c. and orthotopic animal models. **a** Tumour growth curves are shown for DTX or VC treatment groups of PC-3-EpCAM-KD and PC-3-EpCAM-scr s.c. mouse model. Significant differences were seen between PC-3-EpCAM-KD-DTX and PC-3-EpCAM-KD-VC groups (*P* < 0.01), PC-3-EpCAM-scr-DTX and PC-3-EpCAM-scr-VC groups (*P* < 0.01), PC-3-EpCAM-KD-VC and PC-3-EpCAM-scr-VC groups (*P* < 0.05), respectively (*n* = 4). **b** The ratios of DTX-treated versus VC-treated tumour volumes (DTX/VC) were calculated and plotted from the start of DTX treatment to the end of experiment in s.c. mouse model. The ratio of PC-3-EpCAM-KD tumour volumes decreased faster in response to DTX, compared to PC-3-EpCAM-scr group (*P* < 0.01, *n* = 4). **c** Representative images of PC-3-EpCAM-KD and PC-3-EpCAM-scr s.c. xenografts (at the end of the experiment, 5 weeks post DTX treatment). **d** Tumour growth curves are shown for DTX or VC groups of PC-3-EpCAM-KD and PC-3-EpCAM-scr orthotopic mouse model. Significant differences were seen between PC-3-EpCAM-KD-DTX and PC-3-EpCAM-KD-VC groups (*P* < 0.01), PC-3-EpCAM-scr-DTX and PC-3-EpCAM-scr-VC groups (*P* < 0.01), PC-3-EpCAM-KD-VC and PC-3-EpCAM-scr-VC groups (*P* < 0.05), respectively (*n* = 4). **e** The ratios of DTX against VC tumour volumes (DTX/VC) were calculated and plotted from the start of DTX treatment to the end of experiment in orthotopic mouse model. The ratio of PC-3-EpCAM-KD tumour volumes decreased faster in response to RT compared to the sham irradiation (*P* < 0.05, *n* = 4). **f** Characteristic images of PC-3-EpCAM-KD and PC-3-EpCAM-scr orthotopic xenografts (at the end of the experiment, 5 weeks post DTX treatment). DTX, docetaxel; KD, knock-down; scr, scrambled shRNA control; VC, vehicle control

In s.c. mouse model, DTX or VC treatment were administered at 4 weeks post cell inoculation. Tumour volumes were plotted against elapse time (Fig. 3a). Significant differences were seen between PC-3-EpCAM-KD-DTX and PC-3-EpCAM-KD-VC groups (*P* < 0.01), PC-3-EpCAM-scr-DTX and PC-3-EpCAM-scr-VC groups (*P* < 0.01), PC-3-EpCAM-KD-VC and PC-3-EpCAM-scr-VC groups (*P* < 0.05), respectively. The ratios of DTX-treated versus VC-treated tumour volumes (DTX/VC) were calculated and plotted (Fig. 3b). Notably, two groups had different responses to DTX treatment: PC-3-EpCAM-KD xenograft responded to the DTX treatment as early as 1 week post treatment, while PC-3-EpCAM-scr started to respond to DTX at 2 weeks post treatment. Significant differences between two groups in response to DTX were first observed at 2 weeks post DTX treatment. Overall, the ratio of PC-3-EpCAM-KD tumour volumes decreased faster in response to DTX, compared to PC-3-EpCAM-scr group (*P* < 0.01), suggesting that the KD of EpCAM can increase the DTX sensitivity of CaP tumours in s.c. mouse model. The representative images for tumour sizes at the end of experiment are shown in Fig. 3c.

In orthotopic mouse model, DTX or VC treatment was given 4 weeks post cell inoculation. Tumour volumes were plotted against elapse time (Fig. 3d). Significant differences were seen between PC-3-EpCAM-KD-DTX and PC-3-EpCAM-KD-VC groups (*P* < 0.01), PC-3-EpCAM-scr-DTX and PC-3-EpCAM-scr-VC groups (*P* < 0.01), PC-3-EpCAM-KD-VC and PC-3-EpCAM-scr-VC groups (*P* < 0.05), respectively. The ratios of DTX versus VC tumour volumes (DTX/VC) were calculated and plotted (Fig. 3e). Overall, the ratio of PC-3-EpCAM-KD tumour volumes decreased faster in response to DTX compared to the VC group (*P* < 0.05), suggesting that the KD of EpCAM can increase the chemosensitivity of CaP tumours in orthotopic mouse model. The representative images of tumour sizes at the end of experiment are shown in Fig. 3f.

### KD of EpCAM increased radiosensitivity in PC-3 s.c. and orthotopic xenograft mouse models

**Fig. 4:**
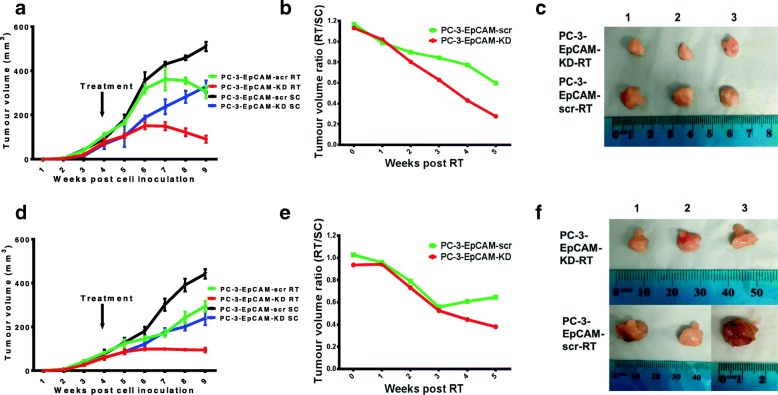
In vivo effects of EpCAM-KD on radiosensitivity in s.c. and orthotopic animal models. **a** Tumour growth curves representing the s.c. mouse model are shown for RT or SC groups of PC-3-EpCAM-KD and PC-3-EpCAM-scr cell lines. Significant differences were seen between PC-3-EpCAM-KD-RT and PC-3-EpCAM-KD-SC groups (*P* < 0.01), PC-3-EpCAM-scr-RT and PC-3-EpCAM-scr-SC groups (*P* < 0.01), PC-3-EpCAM-KD-SC and PC-3-EpCAM-scr-SC groups (*P* < 0.05), respectively (*n* = 4). **b** The ratios of RT versus SC tumour volumes (RT/SC) were calculated and plotted from the start of RT to the end of experiments in s.c. mouse model. The ratio of PC-3-EpCAM-KD tumour volumes decreased faster in response to RT compared to the sham irradiation (*P* < 0.05, *n* = 4). **c** Typical images of PC-3-EpCAM-KD and PC-3-EpCAM-scr s.c. xenografts (at the end of the experiment, 5 weeks post-RT treatment). **d** Tumour growth curves representing the orthotopic mouse model are shown for RT or SC groups of PC-3-EpCAM-KD and PC-3-EpCAM-scr cell lines. Significant differences were seen between PC-3-EpCAM-KD-RT and PC-3-EpCAM-KD-SC groups (*P* < 0.01), PC-3-EpCAM-scr-RT and PC-3-EpCAM-scr-SC groups (*P* < 0.05), PC-3-EpCAM-KD-SC and PC-3-EpCAM-scr-SC groups (*P* < 0.05), respectively (*n* = 4). **e** The ratios of RT versus SC tumour volumes (RT/SC) were calculated and plotted from the start of RT to the end of experiments in orthotopic mouse model. The ratio of PC-3-EpCAM-KD tumour volumes decreased faster in response to RT compared to the sham irradiation (*P* < 0.05, *n* = 4). **f** Representative images of PC-3-EpCAM-KD and PC-3-EpCAM-scr orthotopic xenografts (at the end of the experiment, 5 weeks post RT treatment). KD, knock-down; SC, sham control; RT, radiotherapy; scr, scrambled shRNA control

In s.c. mouse model, RT or SC treatment was given 4 weeks post cell inoculation. Tumour volumes were plotted against elapsed time (Fig. 4a). Significant differences were seen between RT & SC in the KD groups: PC-3-EpCAM-KD-RT and PC-3-EpCAM-KD-SC groups (*P* < 0.01), and between the scr groups: PC-3-EpCAM-scr-RT and PC-3-EpCAM-scr-SC groups (*P* < 0.01), respectively. Additionally, there was a significant difference between SC treatment in KD vs scr: PC-3-EpCAM-KD-SC and PC-3-EpCAM-scr-SC groups (*P* < 0.05). The ratios of RT versus SC tumour volumes (RT/SC) were calculated and plotted (Fig. 4b). Unlike the response of tumours to DTX treatment at an early stage, two groups started to respond to RT from 2 weeks post treatment, showing significant differences in response to RT which were first observed at 2 weeks post RT. Overall, as its response to chemotherapy, the ratio of PC-3-EpCAM-KD tumour volumes decreased faster in response to RT compared to the sham irradiation (*P* < 0.05), suggesting that the KD of EpCAM can increase the radiosensitivity of CaP tumours in s.c. mouse model. The representative images for tumour sizes at the end of experiment are shown in Fig. 4c.

In orthotopic mouse model, RT or SC treatment was given 4 weeks post cell inoculation. Tumour volumes were plotted against elapse time (Fig. 4d). Here also, significant differences were seen between RT and SC in KD and scr: PC-3-EpCAM-KD-RT and PC-3-EpCAM-KD-SC groups (*P* < 0.01), PC-3-EpCAM-scr-RT and PC-3-EpCAM-scr-SC groups (*P* < 0.05), as well as between SC treatment in KD and scr: PC-3-EpCAM-KD-SC and PC-3-EpCAM-scr-SC groups (*P* < 0.05), respectively. The ratios of RT versus SC tumour volumes (RT/SC) were calculated and plotted (Fig. 4e). Starting from 3 weeks post RT, the ratio increased in PC-3-EpCAM-scr group while it continued dropping in PC-3-EpCAM-KD group, suggesting that the tumour suppression effect of RT persisted longer in PC-3-EpCAM-KD group. Overall, as a response to chemotherapy, the ratio of PC-3-EpCAM-KD tumour volumes decreased faster in response to RT compared to the sham irradiation (*P* < 0.05), suggesting that the KD of EpCAM can also increase the radiosensitivity of CaP tumours in orthotopic mouse model. The typical images of tumour sizes at the end of experiment are shown in Fig. 4f.

### KD of EpCAM prolonged the survival of tumour-bearing mice (s.c. model) with and without treatment

**Fig. 5:**
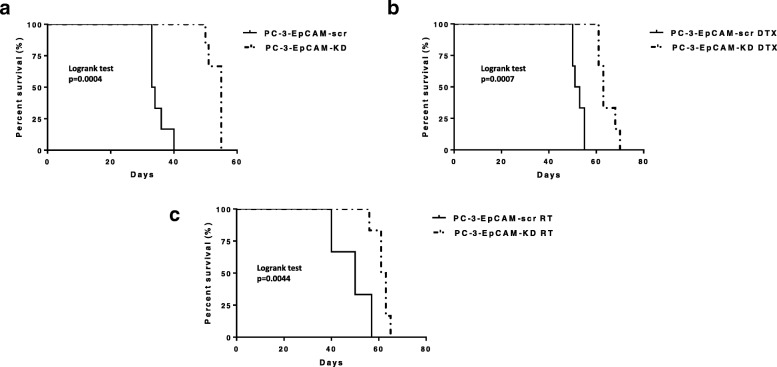
Mouse survival curves after KD of EpCAM and chemo−/radiotherapy in s.c CaP model. **a** Kaplan-Meier survival curves for s.c. tumour-bearing mice demonstrate that KD of EpCAM prolongs mouse survival. *n* = 6, logrank *p* value is shown. **b** Kaplan-Meier survival curves for s.c. tumour-bearing mice that received DTX treatment demonstrate that KD of EpCAM prolongs mouse survival with 40 mg/kg DTX treatment. *n* = 6, logrank *p* value is shown. **c** Kaplan-Meier survival curves for s.c. tumour-bearing mice that received RT demonstrate that KD of EpCAM prolongs mouse survival with 2Gy RT. *n* = 6, logrank p value is shown. DTX, docetaxel, KD, knock-down, RT, radiotherapy

To evaluate the effect of EpCAM on tumour-bearing mice survival, Kaplan-Meier curves demonstrated that KD of EpCAM improved median survival (MS) of tumour-bearing mice (PC-3-EpCAM-KD) by 21.5 days compared with the control group mice (PC-3-EpCAM-scr s.c. xenografts) (HR = 26.94, CI95% 4.317–168.1, *p* = 0.0004) (Fig. 5a). Additionally, KD of EpCAM was found to sensitise the tumour to chemo−/radiotherapy and improved MS of tumour-bearing mice which received DTX (50 mg/kg, single dose, i.p.) by 11 days (HR = 20.95, CI95% 3.599–121.9, *P* = 0.0007) (Fig. 5b), and RT (2Gy/day for 4 days) by 12 days (HR = 11.00, CI95% 2.11–57.36, *P* = 0.0044) (Fig. 5c) when compared with the control group mice (PC-3-EpCAM-scr s.c. xenografts), respectively (Additional file 2: Table S2).

### KD of EpCAM modulates cell proliferation, apoptosis, angiogenesis and therapeutic responses associated with the PI3K/Akt/mTOR signalling pathway

**Fig. 6:**
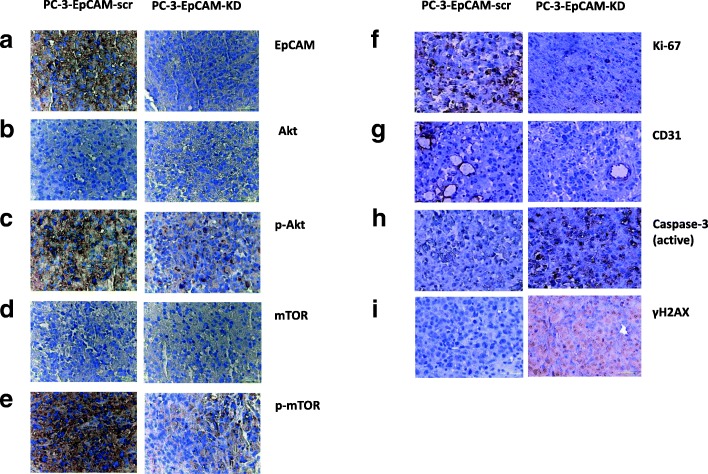
Immunohistochemistry staining of EpCAM, PI3K/mTOR/Akt pathway proteins, Ki-67, CD31 Caspase-3 (active) and γH2AX expressions in s.c. mouse xenografts. **a** Significantly reduced expression of EpCAM was observed after KD of EpCAM in s.c. xenografts. **b**-**e** comparison of EpCAM-KD with EpCAM-scr group: PI3K/Akt/mTOR signalling proteins (p-mTOR and p-Akt) were noticeably downregulated, whereas no obvious changes were seen in mTOR and Akt. **f** Strong expression of Ki-67 was observed in PC-3-EpCAM-scr s.c. xenografts while weak expression of Ki-67 was found inPC-3-EpCAM-KD xenografts. **g** Markedly reduced CD31 expression was seen in PC-3-EpCAM-KD xenografts compared to PC-3-EpCAM-scr xenografts after DTX treatment. **h** After treatment with DTX, an increased level of active Caspase-3 expression was seen in PC-3-EpCAM-KD s.c. xenografts compared to PC-3-EpCAM-scr s.c. xenografts. **i** Following RT, an increased level of γH2AX expression was seen in PC-3-EpCAM-KD s.c. xenografts compared to PC-3-EpCAM-scr s.c. xenografts. Magnification × 400 in all images. DTX, docetaxel; KD, knock-down; RT, radiotherapy; s.c., subcutaneous; scr, scrambled shRNA control

As the PI3K/Akt/mTOR signalling pathway plays a very important role in cancer therapeutic resistance including CaP [14, 15], we investigated whether this pathway is involved in the effects of EpCAM-KD on CaP xenografts using IHC. The results demonstrated that after KD of EpCAM, along with significantly decreased expression of EpCAM, the PI3K/Akt/mTOR signalling proteins (p-mTOR and p-Akt) were also downregulated compared to EpCAM-scr group, whereas no obvious changes were seen in mTOR and Akt between the two groups (Fig. 6, Additional file 3: Table S3).

To further investigate whether KD of EpCAM in CaP cells affects proliferative potential in CaP, tumour sections from s.c. xenografts of NOD/SCID mice were assessed for proliferation using Ki-67 expression. At the end of the experiments, strong expression of Ki-67 was found in PC-3-EpCAM-scr xenograft while a weak to moderate expression of Ki-67 was observed in PC-3-EpCAM-KD xenograft (Fig. 6, Additional file 3: Table S3), suggesting that EpCAM is associated with the proliferation of CaP in vivo.

To study the effects of KD of EpCAM on therapeutic responses, active Caspase-3 and CD31 was examined to assess the tumour response to DTX, while γH2AX was examined to assess the tumour response to RT. At the end of the experiments, there was an increased Caspase-3 (active) and decreased CD31 expression found in the tumour xenografts of PC-3-EpCAM-KD CaP cells compared to PC-3-EpCAM-scr group (Additional file 3: Table S3), suggesting that increased apoptosis (active Caspase-3) and reduced angiogenic activity (CD31) were found in response to DTX treatment after KD of EpCAM. Increased γH2AX expression was found in tumour xenografts of PC-3-EpCAM-KD CaP cells compared to PC-3-EpCAM-scr group after RT (Additional file 3: Table S3), suggesting that sensitisation of CaP to RT could be mediated by KD of EpCAM in vivo. Representative images were shown in Fig. 6.

The staining results showed that EpCAM is associated with cell proliferation, apoptosis, angiogenesis and therapeutic responses in vivo, possibly via regulation of the PI3K/Akt/mTOR signalling pathway, suggesting that this pathway is involved in the regulation of CaP proliferation and chemo−/radioresistance together with EpCAM.

## Discussion

Chemo−/radioresistance remains a big challenge for the current CaP therapy. Therapeutic resistant and refractory CaP cells disseminate to distant organs such as bone and liver via blood and form metastases. Up tp 30% of CaP patients suffered a relapse and developed a PSA recurrence, leading to a more invasive or metastatic disease within 10 years post surgery [16, 17]. Identification of therapeutic targets that are associated with CaP chemo−/radioresistance is crucial for developing novel targeted therapy to overcome CaP therapeutic resistance. In the present study, we investigated whether EpCAM is associated with chemo−/radiotherapy response in CaP animal models and a useful therapeutic target for CaP treatment. We first knocked down EpCAM using shRNA in PC-3 cell line and then established s.c. and orthotopic CaP mouse models using PC-3-EpCAM-KD and PC-3-EpCAM-scr sublines. It was found that KD of EpCAM can significantly reduce tumour growth in both s.c. and orthotopic mouse models, respectively. Our data also indicated that silencing EpCAM increased prostate tumour xenograft sensitivity to chemotherapy and radiation, and significantly prolonged the survival of tumour-bearing mice. Additionally, we showed that EpCAM-regulated tumour development and treatment sensitivity is associated with activation of the PI3K/Akt/mTOR pathway. The findings of in vivo study are consistent with our recent in vitro results [6]. To the best of our knowledge, this is the first study to investigate the roles of EpCAM in CaP development and therapeutic resistance and associated signalling pathway in vivo.

As an oncogene, EpCAM promotes carcinogenesis, cell proliferation and survival, resulting in accelerated tumour growth and metastasis. In our two established xenograft animal models, IHC results also showed that reduction in proliferation (using Ki-67) was associated with KD of EpCAM. Ki-67 is a human nuclear protein that is present during all active phases of the cell cycle but absent from resting cells, making it an excellent marker for measuring the proliferation of cells in human tumours [18]. Since expression of EpCAM is positively related to that of Ki-67, EpCAM may regulate the cell proliferation via cyclin pathway. It was reported that the strength and pattern of EpCAM expression are both positively correlated with the proliferation marker Ki-67, high expression and nuclear localisation of cyclin D1, and Rb phosphorylation in hypopharynx carcinoma in vivo [19], further validating its role in tumorigenesis. Another model of EpCAM signaling mechanism shows that cleaved EpCAM may initiate regulated intramembrane proteolysis that the released cytoplasmic tail of EpCAM is responsible for the induction of transcription of EpCAM target genes [20].

DTX is now the most widely used and first line chemotherapeutic agent for CRPC [21]. In our study, we found that expression of EpCAM is associated with tumour responsiveness to DTX treatment in vivo. DTX achieves its anti-tumour effect by disrupting microtubule dynamics and inducing G2/M cell cycle arrest, which promotes apoptosis through the phosphorylation of antiapoptotic protein Bcl-2 [22]. A recent study by Tayama S, et al. provided evidence that EpCAM overexpression is an independent risk factor for chemoresistance, and correlated with a lower level of cisplatin-induced apoptosis, which is regulated by EpCAM-Bcl-2 axis in ovarian cancer [23]. The EpCAM-Bcl-2 mechanism was further validated in the same study. It was demonstrated that siRNA-mediated EpCAM KD downregulated Bcl-2 expression and upregulated Bax expression whereas transfection of EpCAM reversed the effects [23], providing a rationale for the combination therapy of EpCAM-targeting agents and conventional chemotherapy. In another study, it was found that EpCAM antibody can target EpCAM^+^ chemoresistant leukemia subpopulations after chemotherapy in a mouse model, suggesting EpCAM is a promising novel target for the treatment of leukemia [24]. Interestingly, EpCAM was found to be significantly reduced in docetaxel-resistant PC-3 and DU145 CaP cells, as well as in prostate tissues of patients who received chemotherapeutic treatment with docetaxel [25], which our results (data not shown) were in some degree not in line with. This discrepancy can be explained by the differences in sampling (human patients versus PC-3 mouse xenografts), DOX dosages (60-75 mg/m^2^ versus 50 mg/kg) [26], route of administration (intravenous versus i.p. ) and the post-treatment duration when tumour tissue was obtained (neoadjuvant settings versus 5 weeks).

Over-expression of EpCAM is also related to cancer radiosensitivity. It was demonstrated that higher incidence of intense expression of EpCAM was found in head and neck squamous cell carcinoma (HNSCC) from the hypopharynx, and patients with strong EpCAM expression were associated with local recurrence after primary RT [27]. Kaori et al. reported that down-regulation of EpCAM by siRNA increased the radiosensitivity of ME-180 cervical adenosquamous carcinoma cells [28]. Our results from the in vitro and in vivo study are consistent with Kaorie’s report and indicate that down-regulation of EpCAM expression increased radiosensitivity in CaP. IHC results showed a concomitant upregulation of double-strand breaks marker γH2AX, implying its important role in CaP radiosensitivity, which may help to understand the mechanism by which EpCAM regulates CaP radiosensitivity.

Although multiple previous studies focused on the relationship between EpCAM and survival with patients of CaP, a consensus has not yet been reached [29–31]. In this study, Kaplan-Meier analysis was used to assess the relationship between expression of EpCAM and survival outcomes of CaP tumour-bearing mice. We demonstrated that KD of EpCAM prolonged the survival of tumour-bearing mice with and without chemo−/radiotherapy, suggesting that expression of EpCAM is associated with unfavourable prognosis in CaP.

Activation of the PI3K/Akt/mTOR pathway has been shown to be actively involved in CaP progression [8, 14, 32, 33] and also important in maintenance of a CSC population [34] and promoting EMT in CaP cells [35, 36]. In this study, we have shown that permanent silencing of EpCAM, concomitantly downregulated PI3K/Akt/mTOR signalling pathway proteins and demonstrated it is associated with CaP chemo−/radiosensitivity, suggesting that the activation of this pathway is associated with EpCAM overexpression in vivo. In chemo−/radioresistant recurrent CaP biopsies, we found increased expression of p-AKT and p-mTOR compared with the pre-treatmentbiopsies (unpublished data), further confirming the activation of this signaling pathway in therapeutic resistant CaP patients.

Recent data have demonstrated a pleiotropic role of EpCAM in cancer that is not only limited to regulation of cell-cell adhesion, cell proliferation, migration and signalling [37], but also involved in CSC development and maintenance [8, 38], as well as circulating tumour cells (CTCs) [39]. Mounting data from recent years have indicated that EpCAM is a useful biomarker for the identification and isolation of subsets enriched for CSCs [40, 41], or is associated with the “stemness” in various cancers [23, 42, 43], including CaP [8, 38]. As a result, targeting EpCAM that is responsible for sustaining prostate CSCs and developing treatment resistance might be a promising approach to effectively eradicate prostate CSCs as the putative root of CaP and CaP recurrence.

Based on the close association between EpCAM and CaP development and therapeutic responses, it will be of great clinical significance to use EpCAM in targeted therapy alone or in combination with other conventional therapies in the treatment of CRPC. Possible targeted therapies include antibodies to inactivate EpCAM proteins [44, 45], toxin-conjugated anti-EpCAM antibodies [46], immunotherapy [47], RNAi gene therapies that interfere EpCAM genes [48] as well as EpCAM-conjugated RNA aptamer [49] that can target EpCAM-positive tumour cells with superior affinity and specificity.

## Conclusion

Our data have demonstrated that EpCAM is involved in CaP development, chemo−/radioresistance and prognosis and associated with activation of the PI3K/Akt/mTOR signalling pathway. The findings suggest EpCAM is a promising therapeutic target for overcoming CaP chemo−/radioresistance.

## Additional files

Additional file 1: **Table S1.** List of antibodies used in Western Blot and immunohistochemistry. (DOCX 14 kb)

Additional file 2: **Table S2.** Survival information of the subcutaneous mouse CaP model. (DOCX 13 kb)

Additional file 3: **Table S3.** The staining intensity of various markers in subcutaneous xenografts CaP model. (DOCX 13 kb)

## Abbreviations

Ab: Antibody
BSA: Bovine serum albumin
CaP: Prostate cancer
CRPC: Castration-resistant prostate cancer
CSC: Cancer stem cell
CTC: Circulating tumour cell
DAB: 3,3′ diaminobenzidine
DPBS: Dulbecco’s phosphate-buffered saline
DTX: Docetaxel
ECL: Enhanced chemiluminescence
FBS: Fetal bovine serum
HNSCC: Head and neck squamous cell carcinoma
IHC: Immunohistochemistry
KD: Knockdown
MAb: Monoclonal antibody
MTD: Maximum tolerance dose
PVDF: Polyvinylidene difluoride
RP: Radical prostatectomy
RT: Radiotherapy
s.c.: Subcutaneous
SC: Sham control
SD: Standard deviation
shRNA: Short hairpin RNA
VC: Vehicle control
