# Supplementary material for: Epithelial cell adhesion molecule (EpCAM) is involved in prostate cancer chemotherapy/radiotherapy response in vivo
Source: BMC Cancer. 2018 Nov 12;18:1092. doi: 10.1186/s12885-018-5010-5 (PMC6233586; doi:10.1186/s12885-018-5010-5)
Supplement: Supplementary file 1 — Table S1. List of antibodies used in Western Blot and immunohistochemistry. (DOCX 14 kb) [file 12885_2018_5010_MOESM1_ESM.docx]

Table S1 Antibodies used in WB and IHC

| **Antibody** | **Source** | **Type** | **Dilution** | **Incubation time (min)** | **Temperature** | **Application** | |
| --- | --- | --- | --- | --- | --- | --- | --- |
| **Rabbit anti-human EpCAM** | Abcam | PAb | 1:1000(WB)  1:100(IHC) | O/N | 4°C | | WB, IHC |
| **Rabbit anti-human Akt** | Abcam | PAb | 1:100 (IHC) | O/N | 4°C | | IHC |
| **Rabbit anti-human p-Akt** | Abcam | PAb | 1:100 (IHC) | O/N | 4°C | | IHC |
| **Rabbit anti-human mTOR** | Cell Signaling | MAb | 1:100 (IHC) | O/N | 4°C | | IHC |
| **Rabbit anti-human p-mTOR** | Cell Signaling | MAb | 1:100 (IHC) | O/N | 4°C | | IHC |
| **Mouse anti-human β-tubulin** | Sigma-Aldrich | MAb | 1:5000 (WB) | O/N | 4°C | | IHC |
| **Goat anti-rabbit IgG-HRP** | Santa Cruz Biotechnology | IgG | 1:2500 (WB) | 60 | r/t | | WB |
| **Goat anti-mouse IgG-HRP** | Santa Cruz Biotechnology | IgG | 1:2500 (WB) | 60 | r/t | | WB |
| **Rabbit anti-human Ki-67** | Abcam | MAb | 1:100 (IHC) | O/N | 4°C | | IHC |
| **Rabbit anti-human Caspase-3 (active)** | Abcam | MAb | 1:100 (IHC) | O/N | 4°C | | IHC |
| **Rat anti-mouse CD31** | BD Biosciences | PAb | 1:50 (IHC) | O/N | 4°C | | IHC |
| **Rabbit anti-human γH2AX** | Abcam | PAb | 1:100 (IHC) | O/N | 4°C | | IHC |
| **Rabbit anti-mouse IgG-HRP** | Dako Cytomation | PAb | 1:200 (IHC) | 45 | r/t | | IHC |
| **Goat anti-rabbit IgG-HRP** | Dako Cytomation | PAb | 1:200 (IHC) | 45 | r/t | | IHC |
| **Rabbit anti-rat IgG-Biotinylated** | Dako Cytomation | PAb | 1:300 (IHC) | 45 | r/t | | IHC |

HRP, horseradish peroxide; IF, immunofluorescence; MAb, monoclonal antibody; min: minutes; O/N, overnight; PAb, polyclonal antibody; r/t, room temperature; WB, Western blot; IHC, Immunohistochemistry
