# Supplementary material for: Epithelial cell adhesion molecule (EpCAM) is involved in prostate cancer chemotherapy/radiotherapy response in vivo
Source: BMC Cancer. 2018 Nov 12;18:1092. doi: 10.1186/s12885-018-5010-5 (PMC6233586; doi:10.1186/s12885-018-5010-5)
Supplement: Supplementary file 2 — Table S2. Survival information of the subcutaneous mouse CaP model. (DOCX 13 kb) [file 12885_2018_5010_MOESM2_ESM.docx]

Table S2 Survival information of the subcutaneous mouse CaP model

| Statistics | PC-3-EpCAM-scr vs  PC-3-EpCAM-KD | PC-3-EpCAM-scr-DTX vs PC-3-EpCAM-KD-DTX | PC-3-EpCAM-scr-RT vs PC-3-EpCAM-KD-RT |
| --- | --- | --- | --- |
| MS | 33.5d vs 55d | 52d vs 63d | 50d vs 62d |
| HR | 26.94 | 20.95 | 11.00 |
| 95% CI | 4.317 to 168.1 | 3.599 to 121.9 | 2.110 to 57.36 |
| P | 0.0004 | 0.0007 | 0.0044 |

CI, confidence interval; HR, hazard ratio; MS, median survival.
