# Supplementary material for: Epithelial cell adhesion molecule (EpCAM) is involved in prostate cancer chemotherapy/radiotherapy response in vivo
Source: BMC Cancer. 2018 Nov 12;18:1092. doi: 10.1186/s12885-018-5010-5 (PMC6233586; doi:10.1186/s12885-018-5010-5)
Supplement: Supplementary file 3 — Table S3. The staining intensity of various markers in subcutaneous xenografts CaP model. (DOCX 13 kb) [file 12885_2018_5010_MOESM3_ESM.docx]

Table S3 The intensity staining of EpCAM, PI3K/mTOR/Akt pathway proteins, Ki-67, CD31, Caspase-3 (active) and γH2AX in tumour subcutaneous xenografts from PC-3-EpCAM-KD and PC-3-EpCAM-scr CaP model by IHC

| **Marker** | **Treatment** | **PC-3-EpCAM-KD*** | **PC-3-EpCAM-scr** |
| --- | --- | --- | --- |
| **EpCAM** | - | + | +++ |
| **Akt** | - | + | + |
| **p-Akt** | - | ++ | +++ |
| **mTOR** | - | + | + |
| **p-mTOR** | - | + | +++ |
| **Ki-67** | - | + | ++ |
| **CD31** | DTX | 1-3/hpf | 5-8/hpf |
| **Caspase-3(active)** | DTX | +++ | + |
| **γH2AX** | RT | +++ | + |

All sections were prepared at the end of experiments. Abbreviations: 2, negative; +, weak; ++, moderate; +++, strong. *indicates that obvious difference was found between PC-3-EpCAM-KD and PC-3-EpCAM-scr.
